# An Extension to Deng’s Entropy in the Open World Assumption with an Application in Sensor Data Fusion

**DOI:** 10.3390/s18061902

**Published:** 2018-06-11

**Authors:** Yongchuan Tang, Deyun Zhou, Felix T. S. Chan

**Affiliations:** 1 School of Electronics and Information, Northwestern Polytechnical University, Xi’an 710072, China; dyzhou@nwpu.edu.cn; 2 Department of Industrial and Systems Engineering, The Hong Kong Polytechnic University, Hong Kong, China

**Keywords:** Dempster-Shafer evidence theory (DST), uncertainty measure, open world, closed world, Deng entropy, extended belief entropy, sensor data fusion

## Abstract

Quantification of uncertain degree in the Dempster-Shafer evidence theory (DST) framework with belief entropy is still an open issue, even a blank field for the open world assumption. Currently, the existed uncertainty measures in the DST framework are limited to the closed world where the frame of discernment (FOD) is assumed to be complete. To address this issue, this paper focuses on extending a belief entropy to the open world by considering the uncertain information represented as the FOD and the nonzero mass function of the empty set simultaneously. An extension to Deng’s entropy in the open world assumption (EDEOW) is proposed as a generalization of the Deng’s entropy and it can be degenerated to the Deng entropy in the closed world wherever necessary. In order to test the reasonability and effectiveness of the extended belief entropy, an EDEOW-based information fusion approach is proposed and applied to sensor data fusion under uncertainty circumstance. The experimental results verify the usefulness and applicability of the extended measure as well as the modified sensor data fusion method. In addition, a few open issues still exist in the current work: the necessary properties for a belief entropy in the open world assumption, whether there exists a belief entropy that satisfies all the existed properties, and what is the most proper fusion frame for sensor data fusion under uncertainty.

## 1. Introduction

Uncertain information processing plays a key role in complex systems of many fields such as sensor networks [1,2], pattern recognition [3,4], decision-making [5,6], supply chain network management [7,8], complex network [9] and target tracking [10,11]. Uncertain information may come from sensors with different credibilities and experts’s subjective judgement. The heterogeneous sources and reliable degree increase the complexity and uncertainty of information process. The Dempster-Shafer evidence theory (DST) [12,13] has a promising efficiency in uncertain information processing such as information fusion [14,15]. However, there are still a few open issues in the DST framework that need further study. Firstly, the approaches of managing the conflicting belief masses still needs further refining [16,17]. Secondly, the reasonable ways of generating the mass functions for the practical applications [18,19]. Thirdly, uncertainty quantification with the possible measures in the DST framework [20,21], and the necessary properties a new belief entropy should obey [22,23,24]. Fourthly, rules of combining the body of evidence vary under different circumstances [25,26,27]. Inspired by the open world assumption in [28,29,30], this paper focus on designing an uncertainty measure for the open world in the DST framework.

Uncertainty measure for belief structures is a hot topic for uncertain information processing in DST framework [31]. Many uncertainty measures are derived from Shannon entropy, including Hohle’s confusion measure [32] which is based on the mass function and the belief function of a proposition, Yager’s dissonance measure [33] which is based on the mass function and the plausibility function of a proposition, Dubois and Prade’s weighted Hartley entropy [34] which is based on the mass function of a proposition and the corresponding element number. Both Klir & Ramer’s discord measure [35] and Klir & Parviz’s strife measure [36] are based on two mass functions of different propositions as well as their element number. Recently, some new uncertainty measures emerge, including the general formulation for second-order uncertainties proposed by Yager [37], the maximum likelihood estimation proposed by Denoeux [38], the non–conflicting parts-based conflict measure in belief functions proposed by Daniel [39], Deng entropy proposed by Deng [21] as well as its modification by Tang et al. [40] and uncertainty measure based on interval probabilities [41,42]. According to [21], Deng entropy shows some advantages in some cases in comparison with some other uncertainty measures, and it has been applied in real applications such as the fault diagnosis [43], decision making [44] and sensor data fusion [45]. However, we noticed that all the aforementioned research works handle the uncertainty measure of uncertain information in the closed world, the uncertainty environment in the open world, where there are more sources of uncertainty, has been ignored. Inspired by some research works, especially for the concept of the open world in [28,29,30], we argue that the uncertainty measure in the open world should be different from that in the closed world because the circumstance of uncertainty is different. In the open world assumption of the DST framework, the uncertainty exists in the information expressed by (1) the mass functions of focal elements; (2) the nonzero mass function of the empty set and (3) the possible incompleteness of the frame of discernment (FOD). In the previous uncertainty measures, the uncertain information expressed by the mass function of empty set and the possible incompleteness of the FOD is ignored; which is the reason of this work.

In order to measure the uncertainty of belief structures in the open world, an extended uncertainty measure named the extension to Deng’s entropy in the open world assumption (EDEOW) is proposed. EDEOW is based on the Deng entropy and it can be regarded as a generalization of the Deng entropy. By handling the uncertain information represented by the mass function of the empty set and the uncertain FOD of DST in the open world, EDEOW has the capability of measuring uncertain degree of belief structures for the open world assumption. To verify the usefulness and applicability of the extended belief entropy, some numerical examples and an application on sensor data fusion are presented and discussed in this paper. It should be noticed that a few open issues still exist in the current work, for example, (1) what are the necessary properties for a belief entropy in the open world assumption; (2) whether there exists a belief entropy that satisfies all the existingproperties in the closed world; and (3) what is the proper fusion frame for sensor data fusion under uncertainty.

This rest of this paper is organized as follows. The preliminaries are introduced in Section 2. In Section 3, the EDEOW for uncertainty measure of belief functions is proposed, as well as some illustrative numerical examples. The EDEOW-based information fusion approach and its application in sensor data fusion is presented in Section 4. In Section 5, some open issues for future research related to the work are discussed. Section 6 draws the conclusion of this paper.

## 2. Preliminaries

The background of Dempster-Shafer evidence theory (DST), Shannon entropy and Deng entropy are introduced.

### 2.1. Dempster-Shafer Evidence Theory

Some basic definitions in DST are presented as follows [12,13].

**Definition** **1.** *Assume that $\Omega =\left\{\theta_{1},\theta_{2},\ldots ,\theta_{i},\ldots ,\theta_{N}\right\}$ is a nonempty set with N mutually exclusive and exhaustive events, *Ω* is the frame of discernment (FOD). The power set of *Ω* consists of $2^{N}$ elements denoted as follows:*

(1) $$2^{\Omega }=\left\{\begin{array}{c} \emptyset ,\left\{\theta_{1}\right\},\left\{\theta_{2}\right\},\ldots ,\left\{\theta_{N}\right\},\left\{\theta_{1},\theta_{2}\right\},\\ \ldots ,\left\{\theta_{1},\theta_{2},\ldots ,\theta_{i}\right\},\ldots ,\Omega \end{array}\right\}.$$

**Definition** **2.** *A mass function m is a mapping from the power set $2^{\Omega }$ to the interval [0,1]. m satisfies:*

(2) $$m\left(\emptyset \right)=0,\sum_{A\in \Omega }m\left(A\right)=1.$$

*If $m\left(A\right)>0$, then A is called a focal element. $m\left(A\right)$ indicates the support degree of the evidence on the proposition A.*

**Definition** **3.** *A body of evidence (BOE), also known as a basic probability assignment (BPA) or basic belief assignment (BBA), is defined as the focal sets and the corresponding mass functions:*

(3) $$\left(ℜ,m\right)=\left\{\langle A,m\left(A\right)\rangle :A\in 2^{\Omega },m\left(A\right)>0\right\},$$

*where *ℜ* is a subset of the power set $2^{\Omega }$.*

**Definition** **4.** *A BPA m can also be represented by the belief function Bel or the plausibility function Pl, defined as follows:*

(4) $$Bel\left(A\right)=\sum_{\emptyset \neq B\subseteq A}m\left(B\right),Pl\left(A\right)=\sum_{B\cap A\neq \emptyset }m\left(B\right).$$

**Definition** **5.** *In Dempster-Shafer evidence theory (DST), two independent mass functions $m_{1}$ and $m_{2}$ can be fused with Dempster’s rule of combination:*

(5) $$m\left(A\right)=\left(m_{1}⊕m_{2}\right)\left(A\right)\;=\frac{1}{1-k}\sum_{B\cap C=A}m_{1}\left(B\right)m_{2}\left(C\right),$$

*where k is a normalization factor defined as follows:*

(6) $$k=\sum_{B\cap C=\emptyset }m_{1}\left(B\right)m_{2}\left(C\right).$$

It should be noted that the classical definitions of DST are defined in the closed world. In the open world assumption, Dempster’s rule of combination is extended and named as the generalized combination rule (GCR) by Deng in [30].

**Definition** **6.** *In [30], the fusion result of two empty sets is defined as $\emptyset_{1}\cap \emptyset_{2}=\emptyset$, which means that the intersection between two empty sets is still an empty set. Given two BPAs ($m_{1}$ and $m_{2}$), the generalized combination rule (GCR) is defined as follows:*

(7) $$\begin{array}{c} m\left(A\right)=\frac{\left(1-m\left(\emptyset \right)\right)\sum_{B\cap C=A}m_{1}\left(B\right)\cdot m_{2}\left(C\right)}{1-K},\\ K=\sum_{B\cap C=\emptyset }m_{1}\left(B\right)\cdot m_{2}\left(C\right),\\ m\left(\emptyset \right)=m_{1}\left(\emptyset \right)\cdot m_{2}\left(\emptyset \right),\\ m\left(\emptyset \right)=\begin{array}{ccc} 1 & iff & K=1 \end{array}. \end{array}$$

### 2.2. Shannon Entropy

As the information entropy for uncertainty measure, Shannon entropy has been applied and generalized in many areas such as complexity network [46,47,48].

**Definition** **7.** *Shannon entropy is defined as [49]:*

(8) $$H=-\sum_{i=1}^{N}p_{i}\log_{b}p_{i},$$

*where N is the number of basic states, $p_{i}$ is the probability of state i, $p_{i}$ satisfies $\sum_{i=1}^{N}p_{i}=1$.*

If the unit of information is bit, then b=2. In this case, Shannon entropy is:

(9) $$H=-\sum_{i=1}^{N}p_{i}\log_{2}p_{i}.$$

### 2.3. Deng Entropy

As an extension of Shannon entropy in the framework of DST, Deng entropy is proposed in [21]. Some properties and behaviors are discussed in [21,24]. The application of Deng entropy can be found in [45,50].

**Definition** **8.** *In FOD X, Deng entropy, denoted as $E_{d}$, is defined as:*

(10) $$E_{d}\left(m\right)=-\sum_{A\subseteq X}m\left(A\right)\log_{2}\frac{m\left(A\right)}{2^{\left|A\right|}-1},$$

*where $\left|A\right|$ denotes the cardinality of the proposition A.*

According to [21], the Deng entropy has some advantages in some cases in comparison with some other uncertainty measures in Table 1.

However, Equation (10) will be unavailable if $\left|A\right|=0$. Thus, the uncertainty measure in the closed world of the DST framework should be extended. In the open world assumption [19,30,52,53], the uncertain information represented by the nonzero mass function of the empty set and the incomplete FOD should be handled properly and cautiously.

## 3. New Uncertainty Measure in the Open World

In the DST framework, the uncertain information is modelled not only by mass functions, the FOD is also an important source of uncertainty [40]. In addition, in the open world assumption, the mass value of the empty set may not be zero, which also indicates the incompleteness of the FOD [30]. With this background, how to measure the uncertain degree in the open world assumption of the DST framework is a new perspective and an important issue. According to literature review, no existing uncertainty measure addresses this problem, which is the reason for this work.

**Example** **1.** *Consider a set of BPAs with the FOD $X=\left\{a,b\right\}$, the mass functions with nonzero mass value of the empty set:*

(11) $$m\left(\left\{a\right\}\right)=0.5,m\left(\left\{a,b\right\}\right)=0.3,m\left(\emptyset \right)=0.2.$$

It is obvious that the Deng entropy $E_{d}$ in Equation (10) is not available for the uncertainty measure of BPAs in this case. The denominator of the log function with respect to $m\left(\emptyset \right)=0.2$ will be ($2^{0}-1=0$), which is illegal. This is because the Deng entropy is only based on the mass function of the focal element and the cardinality of the corresponding proposition. In the open world assumption, the mass value of empty set may not be zero. In addition, how to define the element number in incomplete FOD is also an open issue. The same question also exists in other uncertainty measures listed in Table 1. The works in [37,38,40,42] also pay no attention to the possible nonzero mass function of the empty set as well as the possible incomplete element number in the FOD. A new uncertainty measure which is extended from the Deng entropy in the closed world, named the extension to Deng’s entropy in the open world assumption, is proposed especially for the problems mentioned above.

### 3.1. An Extension to Deng’s Entropy in the Open World Assumption

**Definition** **9.** *The extension to Deng’s entropy in the open world assumption is defined as follows:*

(12) $$E_{edeow}\left(m\right)=-\sum_{A\subseteq X}m\left(A\right)\log_{2}\frac{m\left(A\right)}{\left(2^{\left(\left|A\right|+\left\lceil m\left(\emptyset \right)\left|X\right|\right\rceil \right)}-1\right)},$$

*where $\left|A\right|$ is the cardinality of the proposition A, X is the FOD, $\left|X\right|$ denotes the certain element number in the FOD, $\left\lceil m\left(\emptyset \right)\left|X\right|\right\rceil$ is proposed to denote the uncertain element number in the FOD with respect to the corresponding proposition (A). ‘  ’ is the symbol of the ceiling function, which means the smallest integer that is no smaller than the independent variable, e.g., $\left\lceil 0.3\right\rceil =1$.*

The extended measure addresses three parts of uncertainty in the DST framework, including the uncertain information expressed by the mass functions of focal elements, the mass function of the empty set and the possible incompleteness of the FOD. In detail, inspired by the existed uncertainty measures and the Deng entropy, the EDEOW handles two aspects of uncertainty according to the following methods:

- In the closed world where $m\left(\emptyset \right)=0$, the uncertainty represented by the mass function $m\left(A\right)$ of the focal element as well as the corresponding cardinality $\left|A\right|$.
- In the open world where $m\left(\emptyset \right)\neq 0$, the nonzero mass function $m\left(\emptyset \right)$ of the empty set can be an indicator of the completeness or incompleteness of the FOD; currently, $\left\lceil m\left(\emptyset \right)\left|X\right|\right\rceil$ is chosen to express this uncertainty.

It should be noted that, in the EDEOW defined in Equation (12), the proposition *A* is no longer limited as a traditional focal element, it can also be an empty set ∅ which means uncertainty in the FOD [30]. In addition, apart from the $\left\lceil m\left(\emptyset \right)\left|X\right|\right\rceil$, there must exist many types of expressions to express the incompleteness of the FOD.

Recall the BPAs in Equation (11), with the EDEOW, the uncertainty degree of the BPAs can be calculated as follows:

(13) $$\begin{array}{c} E_{edeow}\left(m\right)=-0.5\log_{2}\frac{0.5}{2^{1+\left\lceil 0.2\times 2\right\rceil }-1}-0.3\log_{2}\frac{0.3}{2^{2+\left\lceil 0.2\times 2\right\rceil }-1}\\ -0.2\log_{2}\frac{0.2}{2^{0+\left\lceil 0.2\times 2\right\rceil }-1}=3.1202. \end{array}$$

With the proposed EDEOW, the problem in Example 1 can be handled. The BPAs with a nonzero mass function of the empty set can be handled now with the extended measure.

### 3.2. Numerical Example and Discussion

**Example** **2.** *In FOD $X=\left\{a\right\}$, the mass functions are:*

(14) $$m\left(\left\{a\right\}\right)=1,m\left(\emptyset \right)=0.$$

According to the BPAs in Equation (14), the mass value of the empty set is 0, which indicates the BPAs are assigned in the closed world. The uncertain degree with Shannon entropy *H*, Deng entropy $E_{d}$ and the EDEOW $E_{edeow}$ can be calculated respectively as follows:

(15) $$\begin{array}{c} H\left(m\right)=-1\times \log_{2}1=0,\\ E_{d}\left(m\right)=-1\times \log_{2}\frac{1}{2^{1}-1}=0,\\ E_{edeow}\left(m\right)=-1\times \log_{2}\frac{1}{2^{\left(1+0\right)}-1}=0. \end{array}$$

Obviously, the mass function $m\left(\left\{a\right\}\right)=1$ assigns a belief of 100% on the proposition $\left\{a\right\}$, which means the uncertain degree of the proposition is 0. In this case, the measuring result of the EDEOW is consistent with that of Shannon entropy and Deng entropy.

**Example** **3.** *In FOD $X=\left\{a,b,c,d\right\}$, the mass functions are:*

(16) $$m\left(\left\{a\right\}\right)=m\left(\left\{b\right\}\right)=m\left(\left\{c\right\}\right)=m\left(\left\{d\right\}\right)=0.25,\;m\left(\emptyset \right)=0.$$

The mass value of the empty set is 0, the BPAs are assigned in the closed world. The uncertain degree measured by *H*, $E_{d}$ and $E_{edeow}$ can be calculated respectively as follows:

(17) $$\begin{array}{c} H\left(m\right)=\left(-0.25\times \log_{2}0.25\right)\times 4=2.0,\\ E_{d}\left(m\right)=\left(-0.25\times \log_{2}\frac{0.25}{2^{1}-1}\right)\times 4=2.0,\\ E_{edeow}\left(m\right)=\left(-0.25\times \log_{2}\left(\frac{0.25}{2^{\left(1+0\right)}-1}\right)\right)\times 4=2.0. \end{array}$$

According to the measuring results shown in Equations (15) and (17), if a mass function is assigned on the single subset, then the EDEOW can be degenerated to Deng entropy in the closed world. More importantly, the EDEOW satisfies the property of probabilistic consistency if the BPAs are only assigned on the single subset in the closed world. It should be noted that Shannon entropy and Deng entropy are not available if the BPAs are assigned in the open world where the mass value of the empty set is nonzero; as is shown in Example 1 and the following Example 4.

**Example** **4.** *In a changing FOD $\left|X\right|$, consider the mass functions given as follows:*

(18) $$m\left(\left\{1\right\}\right)=0.2,m\left(\left\{2\right\}\right)=0.3,m\left(\emptyset \right)=0.5.$$

The mass value of the empty set is 0.5, the BPAs are assigned in the open world assumption. The uncertain degree measured by *H*, $E_{d}$ and $E_{edeow}$ are presented in Table 2. Calculation results show that Shannon entropy $E_{d}$ cannot reflect the changes of the cardinality in the FOD $\left|X\right|$ (even if we treat the empty set ∅ as an uncertain proposition with nonzero set to make this function applicable in this case), while the Deng entropy is not applicable in this case for the reason that $m\left(\emptyset \right)\neq 0$. Only the EDEOW can successfully express the enlarging in the FOD as the value of $E_{edeow}\left(m\right)$ increases with the increasing of the $\left|X\right|$.

In the following example adopted from [21], the EDEOW is compared with some other uncertainty measures in the DST framework including Deng entropy $E_{d}$, Yager’s dissonance measure $E_{Y}$, Dubois &Prade’s weighted Hartley entropy $E_{DP}$, Hohle’s confusion measure $C_{H}$, Klir & Ramer’s discord measure $D_{KR}$, Klir & Parviz’s strife measure $S_{KP}$ and George & Pal’s total conflict measure $TC_{GP}$.

**Example** **5.** *In the FOD $X=\left\{1,2,\ldots ,14,15\right\}$, 15 certain elements are denoted as element 1, 2, …, 14, and 15. The mass functions are as follows:*

(19) $$m\left(\left\{6\right\}\right)=0.05,m\left(\left\{3,4,5\right\}\right)=0.05,m\left(Y\right)=0.8,m\left(X\right)=0.1.$$

The element number in the proposition *Y* changes from 0 to 14, as is shown in Table 3. If the element number of *Y* is 0, which means *Y* is an empty set and the FOD may be incomplete, the BPAs are assigned in the open world assumption. In this case, the uncertainty measures $E_{d}$, $E_{Y}$, $E_{DP}$, $C_{H}$, $D_{KR}$, $S_{KP}$ and $TC_{GP}$, which are defined in the closed world is not applicable (N/A). Mathematically, $E_{Y}$ and $C_{H}$ can be applied to calculate the uncertain degree if and only if the constraint of “BPAs are for focal element” is ignored which means a possible modification of the definition of Yager’s dissonance measure $E_{Y}$ and Hohle’s confusion measure $C_{H}$. If the element number of *Y* changes from 1 to 14, then all the uncertainty measures presented in the Section Preliminaries are available for measuring the uncertain degree. The uncertain degree of the BPAs with different uncertainty measures are presented in Table 3, where there is a large discrepancy among the values of the uncertainty measures especially for the proposition *Y* = ∅. Compared with the analysis in [21], the new changes exist in the nonzero mass value of the empty set. The $E_{edeow}$ is the only proper measure in this case compared with other measures listed in Table 3. Of course, we also believe that there are new proper measures for this case since a new measure is always being proposed, e.g., the new entropy in [23].

**Figure 1:**
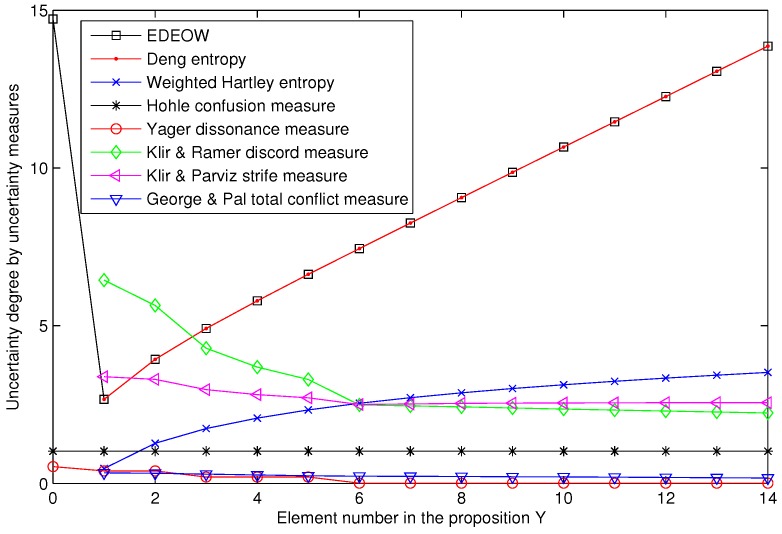
Comparison among different uncertainty measures.

Figure 1 presents the uncertain degree of different uncertainty measures visually. Intuitively, if a big mass value is assigned on the empty set, which means a big uncertain degree in the FOD, in this case, the EDEOW can measure the uncertain degree. It seems that Yager’s dissonance measure $E_{Y}$ and Hohle’s confusion measure $C_{H}$ can be generalized to measure the uncertain degree in the open world assumption where the mass value of the empty set is nonzero. However, Figure 1 shows that the uncertain degree measured by $E_{Y}$ and $C_{H}$ does not increase along with the increasing element number in the proposition *Y*. The $E_{d}$, $E_{DP}$, $D_{KR}$, $S_{KP}$ and $TC_{GP}$ are all not available for uncertainty measure in the open world assumption because of the limitation in the log function of the definitions. Above all, the other uncertainty measures in Table 1 can only be applied in the closed world. Only the EDEOW can successfully measure the uncertainty degree of belief functions in this case. In addition, the EDEOW is identical to Deng entropy in the closed world, which ensures a successful possible extension of the Deng entropy.

### 3.3. A Discussion on the Properties of the Extended Measure

As is discussed in [24], the Deng entropy does not match some of the essential properties for a uncertainty measure in the DST framework. In detail, the Deng entropy satisfies the property of ’probabilistic consistency’, but the properties such as the ’set consistency’, the ’subadditivity’, the ’additivity’ and the ’monotonicity’ are all broken by the Deng entropy. In addition, the range of the Deng entropy is greater than $\left[0,\log_{2}\left|X\right|\right]$. Since the EDEOW is just a simple extension of the Deng entropy, the EDEOW inherits the shortcomings of the Deng entropy with respect to these properties; this should be addressed in the following work.

We noticed that there are new rules of properties defined in a recent research [23], which should be taken into consideration in the ongoing work. Although the extension to Deng’s entropy in the open world assumption only satisfies the property of ’Probabilistic consistency’, we noticed that the newly defined measure in [23] does not satisfy the ’subadditivity’ property, and the distance-based measure in [20] does not satisfy the properties of ’probability consistency’ and ’set consistency’. In short, the property of the belief entropy is still an open issue in the closed world as well as the open world assumption in the DST framework.

## 4. EDEOW-Based Uncertain Information Fusion Approach

**Figure 2:**
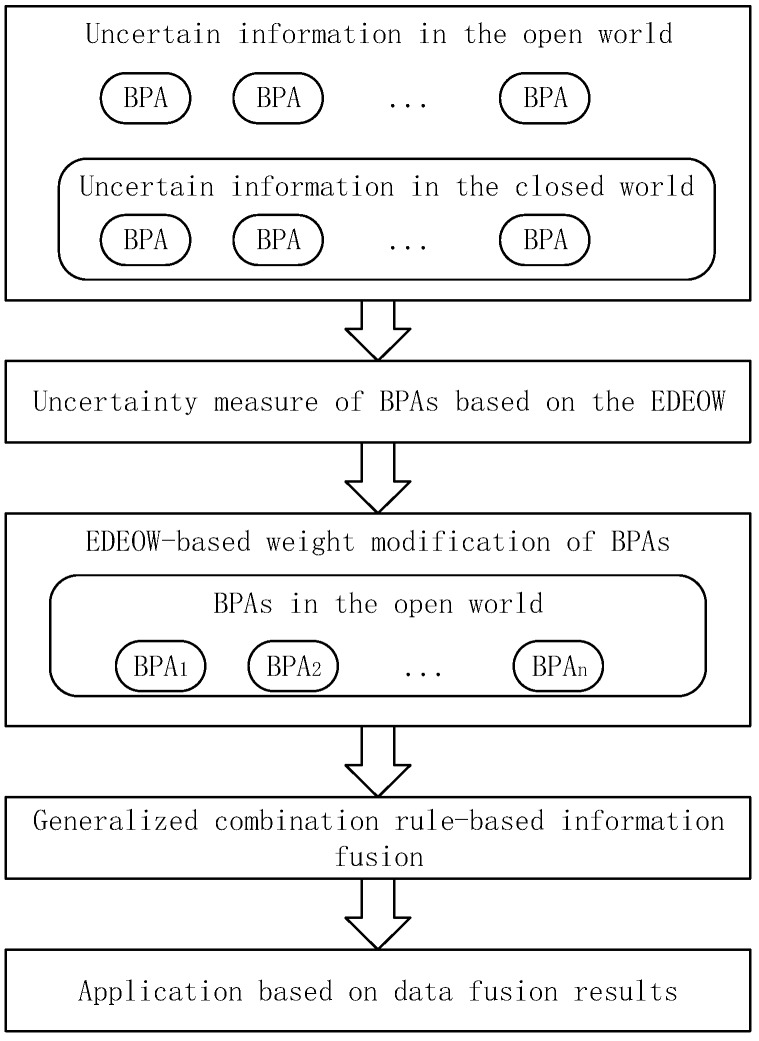
Framework of EDEOW-based uncertain information fusion approach in the open world.

An uncertain information fusion approach based on the EDEOW is proposed to illustrate the usefulness and applicability of the extended measure. The framework of the new approach based on the EDEOW is presented in Figure 2, which is a modification of the methods in [44,54]. Firstly, the uncertain information in the closed world and the open world assumption are modelled as BPAs in DST framework. Then, the EDEOW is adopted to measure the uncertain degree of the BPAs without distinguishing the difference of belief functions in the closed world or the open world assumption, which is accomplished by the advantages that the EDEOW is the extension of an uncertainty measure from the closed world. After that, the uncertain degree measured by the EDEOW is used as the weight of each BPA for modification of the BPAs. Finally, the generalized combination rule in [30] is adopted to combine the BPAs. As a result, applications will be based on the fusion results, such as decision making and fault diagnosis.

The case study in [55] is adopted and modified for verifying the effectiveness of the extended measure, as well as illustrating the EDEOW-based information fusion approach in Figure 2. According to experience and historical data, there are three types of identified fault types in the motor rotor denoted as $F_{1}=\left\{Rotor\;\;unbalance\right\}$, $F_{2}=\left\{Rotor\;\;misalignment\right\}$ and $F_{3}=\left\{Pedestal\;\;looseness\right\}$ respectively. The vibration signal is collected by three acceleration sensors placed in different positions. The acceleration sensors can collect the signals at different frequencies denoted as Freq1, Freq2 and Freq3, the signals will be used as the judgement variables of fault types. The monitoring results of sensors are modelled as BPAs in Table 4 adopted from [55].

For each frequency, the BPAs reported by three sensors are denoted as $m_{s_{1}}\left(\cdot \right)$, $m_{s_{2}}\left(\cdot \right)$ and $m_{s_{3}}\left(\cdot \right)$. {F1,F2,F3} is the FOD of this application in the closed world. Here, in this paper, in order to adapt the experiment data for the application of the extended measure in the open world, the belief functions of {F1,F2,F3} are assumed to be assigned to the empty set ∅, which extends the uncertainty of the FOD from the closed world to the open world. This is reasonable, because there may exist unknown fault types.

### 4.1. Uncertainty Measure of BPAs with EDEOW

In real applications, the reliability of each sensor is unknown. Thus, the uncertain degree of sensor reports should be measured properly. In the DST framework, the belief entropy is proposed for measuring the uncertainty of BPAs. Once the sensor reports are modelled as BPAs, the uncertain degree of sensor reports can be measured based on the EDEOW in Equation (12). For example, for the BPAs of Freq1, the uncertain degree with the EDEOW is calculated as follows:

(20) $$\begin{array}{c} E_{edeow}\left(m_{s_{1}}\right)=-0.8176\log_{2}\frac{0.8176}{2^{1+\left\lceil 0.0268\times 3\right\rceil }-1}\\ -0.0003\log_{2}\frac{0.0003}{2^{1+\left\lceil 0.0268\times 3\right\rceil }-1}\\ -0.1553\log_{2}\frac{0.1553}{2^{2+\left\lceil 0.0268\times 3\right\rceil }-1}\\ -0.0268\log_{2}\frac{0.0268}{2^{0+\left\lceil 0.0268\times 3\right\rceil }-1}\\ =2.5306,\\ E_{edeow}\left(m_{s_{2}}\right)=-0.5658\log_{2}\frac{0.5658}{2^{2+\left\lceil 0.3687\times 3\right\rceil }-1}\\ -0.0009\log_{2}\frac{0.0009}{2^{2+\left\lceil 0.3687\times 3\right\rceil }-1}\\ -0.0646\log_{2}\frac{0.0646}{2^{2+\left\lceil 0.3687\times 3\right\rceil }-1}\\ -0.3687\log_{2}\frac{0.3687}{2^{0+\left\lceil 0.3687\times 3\right\rceil }-1}\\ =3.6877,\\ E_{edeow}\left(m_{s_{3}}\right)=-0.2403\log_{2}\frac{0.2403}{2^{2+\left\lceil 0.7452\times 3\right\rceil }-1}\\ -0.0004\log_{2}\frac{0.0004}{2^{2+\left\lceil 0.7452\times 3\right\rceil }-1}\\ -0.0141\log_{2}\frac{0.0141}{2^{2+\left\lceil 0.7452\times 3\right\rceil }-1}\\ -0.7452\log_{2}\frac{0.7452}{2^{0+\left\lceil 0.7452\times 3\right\rceil }-1}\\ =4.0040. \end{array}$$

The uncertain degree of Freq2 and Freq3 can also be calculated by Equation (12). The results are presented in Table 5.

### 4.2. EDEOW-Based Modification of BPAs

The EDEOW of each BPA is used as the weight factor of each sensor report. With a process of normalization, the weight of each BPA in Freq1 is calculated as follows:

(21) $$\begin{array}{c} w_{S_{1}}=E_{edeow}\left(m_{s_{1}}\right)/\sum_{i=1}^{3}E_{edeow}\left(m_{s_{i}}\right)=0.2476,\\ w_{S_{2}}=E_{edeow}\left(m_{s_{2}}\right)/\sum_{i=1}^{3}E_{edeow}\left(m_{s_{i}}\right)=0.3608,\\ w_{S_{3}}=E_{edeow}\left(m_{s_{3}}\right)/\sum_{i=1}^{3}E_{edeow}\left(m_{s_{i}}\right)=0.3917. \end{array}$$

Similarly, the weight of BPAs in Freq2 and Freq3 can be calculated. After normalization, the weight of each BPA for Freq1, Freq2 and Freq3 is listed in Table 6.

The modification of BPAs for each frequency can be calculated with the following equation:

(22) $$m_{w}\left(\cdot \right)=w_{s_{i}}m_{s_{i}}.$$

Based on the normalized weight factor in Table 6, with Equation (22), the modified BPAs of Freq1 is calculated as follows:

(23) $$\begin{array}{c} m_{w}\left(\left\{F2\right\}\right)=0.5006,\\ m_{w}\left(\left\{F3\right\}\right)=0.0005,\\ m_{w}\left(\left\{F1,F2\right\}\right)=0.0673,\\ m_{w}\left(\emptyset \right)=0.4315. \end{array}$$

The modification of BPAs for Freq2 and Freq3 can be calculated with Equation (22). The BPAs after modification of each frequency is shown in Table 7.

### 4.3. Generalized Combination Rule-Based Data Fusion

In the open world assumption, classical Dempster’s rule of combination is not applicable [30]. In this paper, the generalized combination rule in [30] is chosen for data fusion in the proposed approach. Since the original *n* sets of BPAs have been modified as one set of BPAs by EDEOW-based weight factors, the modified BPAs should be fused (n−1) times according to the chosen combination rule.

There are three sets of BPAs before modification. Thus, the modified BPAs should be combined two times with generalized combination rule in Equation (7). For frequency Freq1, the fusion results are shown as follows:

(24) $$\begin{array}{c} m\left(\left\{F2\right\}\right)={\left({\left(m_{w}⊕m_{w}\right)}_{1}⊕m_{w}\right)}_{2}\left(\left\{F2\right\}\right)=0.9181,\\ m\left(\left\{F3\right\}\right)={\left({\left(m_{w}⊕m_{w}\right)}_{1}⊕m_{w}\right)}_{2}\left(\left\{F3\right\}\right)=0.0000,\\ m\left(\left\{F1,F2\right\}\right)={\left({\left(m_{w}⊕m_{w}\right)}_{1}⊕m_{w}\right)}_{2}\left(\left\{F1,F2\right\}\right)=0.0015,\\ m\left(\emptyset \right)={\left({\left(m_{w}⊕m_{w}\right)}_{1}⊕m_{w}\right)}_{2}\left(\emptyset \right)=0.0803. \end{array}$$

The BPAs of Freq2 and Freq3 are also fused three times with the generalized combination rule, the results are shown in Table 8.

With the fusion results shown in Table 8, F2 significantly has the highest support degree among all the frequencies, therefore, we can judge that the fault type is F2. The experiment results are consistent with [54,55], which verifies the effectiveness of the EDEOW. In addition, the proposed method has a higher support degree on the recognized fault type F2 than that in [54,55], which is good for decision-making by engineers in real applications.

## 5. Open Issues for Future Work

There is no universally accepted measure for uncertainty quantification in the DST framework. Many new measures are still being proposed within one year [23,56]. To match the open world assumption [19,30,52,53], an extended measure for quantification of uncertain degree in the DST framework is proposed in this paper. It should be noted that the extended measure is a simple extension of the Deng entropy. A lot of open issues exist in the extended measure as well as the other measures for the open world assumption in the DST framework.

The first one exists in the scope of the uncertainty measures in the DST framework. According to the current research, we find that the theory of belief entropy or uncertainty measures in the DST is still not solid and needs further deep research. We suggest that the following research work on this topic should take into consideration the open world assumption.

The second open issue exists in the properties of the extended measure, which is a shortcoming inherited from the Deng entropy. According to the research work in [24], the Deng entropy only satisfies the property of probabilistic consistency with respect to the five requirements for a total uncertainty measure. The following work should focus on improving the measure or developing a totally new uncertainty measure for the open world assumption by taking into consideration all of the properties discussed in [22,23,24,57].

Thirdly, the following work needs to investigate what happens if the mass on the empty set is not null with different size of the universe because the new measure in the open world must address these two parameters. In addition, what is the meaning of having an entropy measure that changes in accordance with the cardinality of the universe? For instance, for a FOD $\left|X\right|$, we will have the same measure result $E_{edeow}\left(m\right)=0$ for the mass function $m\left(\emptyset \right)=1$ and $m\left(\left\{a\right\}\right)=1$. Currently, we have difficulty answering all of these questions in this simple extended measure.

Fourthly, there are still no universally accepted properties for a belief entropy or uncertainty measure in the closed world and for the open world assumption, which is a big problem for developing a new belief entropy. For example, even the newly defined measure in [23] does not satisfy the ’subadditivity’ property. Another example is that the measure in [20] does not satisfy the properties of ’probability consistency’ and ’set consistency’. We believe that there are new properties that should be obeyed by the measures in the open world assumption.

Finally, in the application of sensor data fusion, fusion frame and combination rule need further study. There are more fusion methods and the combination rules in the research works [25,26,53,58] that need to be investigated cautiously.

## 6. Conclusions

An extended uncertainty measure for belief structures in the open world assumption, named the EDEOW, is proposed in this paper. The extended measure can successfully quantify the uncertain degree of belief structures not only in the closed world, but also in the open world. With the extended measure, more uncertain information in DST framework is taken into consideration while applying information processing, including the possible incomplete FOD and the nonzero mass function of the empty set, of which both are sources of uncertainty in the DST framework in the open world assumption. To verify the usefulness and applicability of the extended measure, the EDEOW is adopted to design a new information fusion approach in the open world circumstance. Numerical examples and the application on the sensor data fusion-based fault diagnosis verify the effectiveness of the proposed method. The limitations and open issues for possible future research are also discussed in this paper.

## Figures and Tables

**Table 1:** Uncertainty measures in DST framework.

Uncertainty Measure	Definition
Hohle’s confusion measure [32]	$C_{H}\left(m\right)=-\sum_{A\subseteq X}m\left(A\right)\log_{2}Bel\left(A\right)$
Yager’s dissonance measure [33]	$E_{Y}\left(m\right)=-\sum_{A\subseteq X}m\left(A\right)\log_{2}Pl\left(A\right)$
Dubois &Prade’s weighted Hartley entropy [34]	$E_{DP}\left(m\right)=\sum_{A\subseteq X}m\left(A\right)\log_{2}\left|A\right|$
Klir & Ramer’s discord measure [35]	$D_{KR}\left(m\right)=-\sum_{A\subseteq X}m\left(A\right)\log_{2}\sum_{B\subseteq X}m\left(B\right)\frac{\left|A\cap B\right|}{\left|B\right|}$
Klir & Parviz’s strife measure [36]	$S_{KP}\left(m\right)=-\sum_{A\subseteq X}m\left(A\right)\log_{2}\sum_{B\subseteq X}m\left(B\right)\frac{\left|A\cap B\right|}{\left|A\right|}$
George & Pal’s total conflict measure [51]	$TC_{GP}\left(m\right)=\sum_{A\subseteq X}m\left(A\right)\sum_{B\subseteq X}m\left(B\right)\left(1-\frac{\left|A\cap B\right|}{\left|A\cup B\right|}\right)$

**Table 2:** Uncertain degree with different measures in Example 4.

Uncertainty Measure	$\left|X\right|=2$	$\left|X\right|=3$	$\left|X\right|=5$	$\left|X\right|=7$	$\left|X\right|=9$
$E_{d}\left(m\right)$	-	-	-	-	-
$H\left(m\right)$	1.4855	1.4855	1.4855	1.4855	1.4855
$E_{edeow}\left(m\right)$	2.2780	3.6816	4.8426	5.9160	6.9512

**Table 3:** The EDEOW $E_{edeow}$, Deng entropy $E_{d}$, Yager’s dissonance measure $E_{Y}$, Dubois & Prade’s weighted Hartley entropy $E_{DP}$, Hohle’s confusion measure $C_{H}$, Klir & Ramer’s discord measure $D_{KR}$, Klir & Parviz’s strife measure $S_{KP}$ and George & Pal’s total conflict measure $TC_{GP}$ with the variable proposition *Y*. (For computing the $E_{Y}$ and $C_{H}$, we treat ’*Y* = ∅’ as a special proposition in this case to compute the corresponding values.)

Proposition	$E_{\mathrm{edeow}}$	$E_{d}$	$E_{Y}$	$E_{\mathrm{DP}}$	$C_{H}$	$D_{\mathrm{KR}}$	$S_{\mathrm{KP}}$	${\mathrm{TC}}_{\mathrm{GP}}$
Y=∅	14.7216	N/A	(0.5312)	N/A	(1.0219)	N/A	N/A	N/A
$Y=\left\{1\right\}$	2.6623	2.6623	0.3952	0.4699	1.0219	6.4419	3.3804	0.3317
$Y=\left\{1,2\right\}$	3.9303	3.9303	0.3952	1.2699	1.0219	5.6419	3.2956	0.3210
$Y=\left\{1,2,3\right\}$	4.9082	4.9082	0.1997	1.7379	1.0219	4.2823	2.9709	0.2943
$Y=\left\{1,\ldots ,4\right\}$	5.7878	5.7878	0.1997	2.0699	1.0219	3.6863	2.8132	0.2677
$Y=\left\{1,...,5\right\}$	6.6256	6.6256	0.1997	2.3274	1.0219	3.2946	2.7121	0.2410
$Y=\left\{1,\ldots ,6\right\}$	7.4441	7.4441	0.0074	2.5379	1.0219	2.4888	2.4992	0.2250
$Y=\left\{1,\ldots ,7\right\}$	8.2532	8.2532	0.0074	2.7158	1.0219	2.4562	2.5198	0.2219
$Y=\left\{1,...,8\right\}$	9.0578	9.0578	0.0074	2.8699	1.0219	2.4230	2.5336	0.2170
$Y=\left\{1,...,9\right\}$	9.8600	9.8600	0.0074	3.0059	1.0219	2.3898	2.5431	0.2108
$Y=\left\{1,\ldots ,10\right\}$	10.6612	10.6612	0.0074	3.1275	1.0219	2.3568	2.5494	0.2037
$Y=\left\{1,\ldots ,11\right\}$	11.4617	11.4617	0.0074	3.2375	1.0219	2.3241	2.5536	0.1959
$Y=\left\{1,\ldots ,12\right\}$	12.2620	12.2620	0.0074	3.3379	1.0219	2.2920	2.5562	0.1877
$Y=\left\{1,\ldots 13\right\}$	13.0622	13.0622	0.0074	3.4303	1.0219	2.2605	2.5577	0.1791
$Y=\left\{1,\ldots ,14\right\}$	13.8622	13.8622	0.0074	3.5158	1.0219	2.2296	2.5582	0.1701

**Table 4:** Data for fault diagnosis modelled as BPAs [55].

		Freq1					Freq2				Freq3	
	{F2}	{F3}	{F1,F2}	**∅**		{F2}	**∅**		{F1}	{F2}	{F1,F2}	**∅**
$m_{s_{1}}\left(\cdot \right)$	0.8176	0.0003	0.1553	0.0268		0.6229	0.3771		0.3666	0.4563	0.1185	0.0586
$m_{s_{2}}\left(\cdot \right)$	0.5658	0.0009	0.0646	0.3687		0.7660	0.2341		0.2793	0.4151	0.2652	0.0404
$m_{s_{3}}\left(\cdot \right)$	0.2403	0.0004	0.0141	0.7452		0.8598	0.1402		0.2897	0.4331	0.2470	0.0302

**Table 5:** Uncertainty measure results of sensor reports based on EDEOW.

$E_{\mathrm{edeow}}\left(\cdot \right)$	Freq1	Freq2	Freq3
$E_{edeow}\left(m_{s_{1}}\right)$	2.5306	3.3024	3.2887
$E_{edeow}\left(m_{s_{2}}\right)$	3.6877	1.9991	3.5804
$E_{edeow}\left(m_{s_{3}}\right)$	4.0040	1.9475	3.5305

**Table 6:** EDEOW-based weight factor of BPAs after normalization.

$w_{S_{i}}$	Freq1	Freq2	Freq3
$w_{S_{1}}$	0.2476	0.4556	0.3162
$w_{S_{2}}$	0.3608	0.2758	0.3443
$w_{S_{3}}$	0.3917	0.2687	0.3395

**Table 7:** Modified mass function based on EDEOW.

		Freq1					bold-italic2				bold-italic3	
	{F2}	{F3}	{F1,F2}	**∅**		{F2}	**∅**		{F1}	{F2}	{F1,F2}	**∅**
$m_{w}\left(\cdot \right)$	0.5006	0.0005	0.0673	0.4315		0.7260	0.2740		0.3104	0.4342	0.2126	0.0427

**Table 8:** Sensor data fusion results with different methods.

		Freq1					Freq2				Freq3	
	{F2}	{F3}	{F1,F2}	**∅**		{F2}	**∅**		{F1}	{F2}	{F1,F2}	**∅**
Jiang et al.’s method [55]	0.8861	0.0002	0.0582	-		0.9621	-		0.3384	0.5904	0.0651	-
Tang et al.’s method [54]	0.8891	0.0003	0.0427	-		0.9784	-		0.3318	0.6332	0.0349	-
The proposed method	0.9181	0.0000	0.0015	0.0803		0.9796	0.0206		0.3303	0.6459	0.0238	0.0001
